# Editorial: Exercise Prescription and Psychological Determinants for Healthy Living

**DOI:** 10.3389/fpsyg.2022.851852

**Published:** 2022-04-26

**Authors:** Matteo Bonato, Jacopo Antonino Vitale, Guillermo Felipe López Sánchez, Roberto Codella

**Affiliations:** ^1^Department of Biomedical Sciences for Health, Università degli Studi di Milano, Milan, Italy; ^2^IRCCS Istituto Ortopedico Galeazzi, Milan, Italy; ^3^Division of Preventive Medicine and Public Health, Department of Public Health Sciences, School of Medicine, University of Murcia, Murcia, Spain; ^4^Department of Endocrinology, Nutrition and Metabolic Diseases, IRCCS MultiMedica, Milan, Italy

**Keywords:** physical activity, exercise prescription, cardiorespiratory fitness, health, exercise adherence

The Frontiers Research Topic titled “*Exercise Prescription and Psychological Determinants for Healthy Living*” aimed at providing evidence of the impact of physical activity (PA) in improving physical fitness and managing chronic diseases/conditions while ensuring mental health.

It is commonly accepted that PA delays all-cause mortality in the general population and reduces the risk of chronic diseases, including cardiovascular diseases, type 2 diabetes mellitus, and, in some cases, even cancer (Garber et al., 2011). Moreover, in recent years there has been a growing interest in investigating the determinants of health-related quality of life (Sartor et al., 2016). Consistently, longitudinal studies have shown improvements in cardiorespiratory fitness, muscle strength, body composition, depression symptoms, and quality of life after PA (Blair et al., 2012).

In this regard, exercise prescription is the systematic and planned execution of tailored PA through which people can improve their cardiorespiratory and muscle fitness, preventing the development of non-communicable diseases (e.g., diabetes, cardiovascular diseases, cancers etc.), and finally optimize their overall health, fitness or feeling of wellbeing. Both the World Health Organization and the American College of Sport Medicine recommend in general 150 min of moderate-intensity activity or 75 min of vigorous-intensity activity per week, or a combination of both (Garber et al., 2011). Indeed, the undertaking of PA remains challenging and the optimal exercise parameters (e.g., mode, intensity, duration, etc.) for the different populations still need to be confirmed.

Nevertheless, long-term adherence to PA, which is key to such earned health and psychological benefits, is still low (Garber et al., 2011). In fact, it has been observed that individualized-tailored behavioral exercise programs can enhance the adoption and short-term adherence to exercise. Exercise programs conducted in diverse populations in a variety of settings have been effective in promoting short-term increases in PA when they are (i) based on health behavioral theoretical constructs; (ii) individually-tailored; (iii) using behavioral strategies such as goal setting, social support, reinforcement, and relapse prevention. Individually-tailored interventions, delivered by various modalities including print, telephone, internet/computer/smartphone applications, and group counseling, can be effective in enhancing exercise adoption. However, they are all best marginally effective for increasing long-term exercise maintenance (Albergoni et al., 2019). Despite the well-documented issues with long-term exercise adherence and exercise dropouts, few data exist regarding the factors associated with maintaining exercise behavior.

The opinion article of Lakicevic et al. highlighted that individuals' PA participation often decreases over time due to a decrease in personal interest and enjoyment. Therefore, in order to prevent the premature dropout from engaging in a PA program, the authors suggest to introduce a reinforcement of intrinsic motivation like varying familiar exercises with a different training routine and/or novel activities (e.g., exergames, smartphone apps etc.) so to arise participants' interest and enjoyment. Physicians, researchers, and practitioners should place a special emphasis on novelty, which is one of the key determinants contributing to PA adherence. In fact, enjoyable PA improves health and wellbeing, and provides continual novel stimulus, leading to improved engagement and adherence over time.

However, the spread of COVID-19 resulted in numerous restrictions on daily living including social distancing, isolation and home lockdown that improved peoples' sedentary behavior. To this regard, the article of Van Luchene et al. highlighted that Belgian population did not share the same perception of the influence of COVID-19 lockdown on the practice of PA. Indeed, their findings suggested that workers, especially students, are more likely to feel the influence of lockdown as positive in terms of their active behavior, unlike retirees who are more likely to feel it more negatively. Therefore, authors suggested that leisure professionals could promote PA and social wellbeing in older people by increasing opportunities at home, by delivering additional online leisure services, volunteering opportunities and social interactions.

Another study during the COVID-19 pandemic by Casali et al. found that elite athletes reduced their training schedule more than non-elite pairs, indicating that these latter may have been more negatively affected by restrictions. Furthermore, authors pointed to gender differences in terms of distress, mental health, and body dissatisfaction experienced during lockdown, with female athletes being more impaired than males in these areas. Finally, mediation models showed that vigorous PA positively affected both mental and physical health during the lockdown. Their findings suggest that while COVID-19 lockdown was taxing for athletes, particularly professionals, those who were able to practice PA were able to exploit exercise benefits during a stressful situation such as COVID-19 lockdown, reporting physical health.

El-Shafei et al. examined, from a biopsychosocial point of view, the combined effect of wearing two different heel heights and of hormonal oscillation throughout different phases of the menstrual cycle on spinopelvic alignment. Authors found that there were no significant changes of kyphotic angle, trunk inclination, and pelvic inclination while wearing low- (2.5 cm) and high-heeled shoes (7 cm) during early follicular, ovulatory, and mid-luteal phases. Considering that high-heeled shoes are traditionally associated with femininity, body image, beauty, and charm, authors concluded that this research could have important biopsychosocial implications that should be explored in detail in future studies.

Finally, Gacek et al. aimed to analyse personality-related determinants of physical activity among Polish and Spanish physical education students. Authors found that positive correlations between PA, extraversion and conscientiousness, as well as negative ones with neuroticism, were demonstrated among Polish and Spanish students, and the moderating impact of the country on the correlation between personality-related dimensions and physical activity.

In conclusion, all highlighted that exercise maintenance and the development of behavioral, holistic theory are needed, to understand not only how assisting individuals in initiating exercise and reducing sedentary behavior, but also how people can maintain an active lifestyle over time.

## Author Contributions

MB wrote the first draft of the present manuscript. JV, GL, and RC contributed to its critical revision. All authors contributed to the article and approved the submitted version.

## Conflict of Interest

The authors declare that the research was conducted in the absence of any commercial or financial relationships that could be construed as a potential conflict of interest.

## Publisher's Note

All claims expressed in this article are solely those of the authors and do not necessarily represent those of their affiliated organizations, or those of the publisher, the editors and the reviewers. Any product that may be evaluated in this article, or claim that may be made by its manufacturer, is not guaranteed or endorsed by the publisher.
